# Neural Representation of Articulable and Inarticulable Novel Sound Contrasts: The Role of the Dorsal Stream

**DOI:** 10.1162/nol_a_00016

**Published:** 2020-08-01

**Authors:** David I. Saltzman, Emily B. Myers

**Affiliations:** University of Connecticut, Storrs, CT; University of Connecticut, Storrs, CT

**Keywords:** speech perception, fMRI, multivoxel pattern analysis, speech articulation, dorsal stream, phonetics

## Abstract

The extent that articulatory information embedded in incoming speech contributes to the formation of new perceptual categories for speech sounds has been a matter of discourse for decades. It has been theorized that the acquisition of new speech sound categories requires a network of sensory and speech motor cortical areas (the “dorsal stream”) to successfully integrate auditory and articulatory information. However, it is possible that these brain regions are not sensitive specifically to articulatory information, but instead are sensitive to the abstract phonological categories being learned. We tested this hypothesis by training participants over the course of several days on an articulable non-native speech contrast and acoustically matched inarticulable nonspeech analogues. After reaching comparable levels of proficiency with the two sets of stimuli, activation was measured in fMRI as participants passively listened to both sound types. Decoding of category membership for the articulable speech contrast alone revealed a series of left and right hemisphere regions *outside* of the dorsal stream that have previously been implicated in the emergence of non-native speech sound categories, while no regions could successfully decode the inarticulable nonspeech contrast. Although activation patterns in the left inferior frontal gyrus, the middle temporal gyrus, and the supplementary motor area provided better information for decoding articulable (speech) sounds compared to the inarticulable (sine wave) sounds, the finding that dorsal stream regions do not emerge as good decoders of the articulable contrast alone suggests that other factors, including the strength and structure of the emerging speech categories are more likely drivers of dorsal stream activation for novel sound learning.

## INTRODUCTION

Whether an infant learning her first language, or an adult learning his fifth language, in language acquisition the learner must learn to perceive as well as produce new speech sounds. In typical language acquisition, these two processes (perceptual and articulatory learning) develop in tandem, although not necessarily on the same timeline (Tsao, Liu, & Kuhl, 2004). However, in laboratory conditions, we know that individuals can learn to distinguish complex auditory speech and nonspeech categories without learning to produce these same categories. At issue is the degree to which articulatory information associated with speech is recruited even in the absence of articulatory learning. That is, when one learns a new phonetic category without learning to produce the same category, are speech motor representations recruited?

Models of speech perception like the motor theory of speech (Liberman, Cooper, Shankweiler, & Studdert-Kennedy, 1967) and subsequent direct realist approaches (Best, 1995; Fowler, 1986), make explicit predictions about the formative role of articulatory codes (i.e., the gestures made by the articulators to create a speech sound) in speech perception. In these models, motor or articulatory representations are the objects of perception, and must be acquired and then accessed to achieve robust comprehension (see Galantucci, Fowler, & Turvey, 2006 for a review). Thus, learning to discriminate between a pair of speech sounds like /b/ and /d/ (which are similar acoustically) requires that the listener access information the articulatory gestures used to produce those sounds.

Though not explicitly linked, the dual stream model of speech processing (Hickok & Poeppel, 2007) makes biological predictions that also implicate motor systems not in speech sound *perception*, but in speech sound *learning*. Specifically, the dorsal stream pathway, consisting of such brain regions as the inferior frontal gyrus (IFG), the pre-motor cortex, and the temporal-parietal junction, is claimed to play a critical role in auditory-motor integration and serves as a “neural mechanism that both codes and maintains instances of speech sounds,” which can then be later used to aid in production as well as in perception (Hickok & Poeppel, 2007, p. 399). According to this view, during the learning of new speech sounds, listeners code the incoming articulatory gestures in dorsal stream areas in preparation for future speech output (Hickok & Buchsbaum, 2003). Notably, once speech sound representations have been learned, dorsal stream activation is no longer thought to be required during perception-only tasks.

The role of the dorsal stream during speech perception (for established speech categories and newly learned ones) has been a matter of significant debate, as has the role of articulatory codes during speech perception in general (e.g., Lotto, Hickok, & Holt, 2009; Schomers & Pulvermüller, 2016; Wilson, 2009; Scott, McGettigan, & Eisner, 2009). If articulatory codes are accessed as a matter of course during perception of speech categories, it should follow that the encompassed brain regions are sensitive to differences in articulation between speech sounds during speech perception, especially during speech sound learning (see Meister, Wilson, Deblieck, Wu, & Iacoboni, 2007; Wilson & Iacoboni, 2006; Wilson, Saygin, Sereno, & Iacoboni, 2004). While evidence for sensitivity to place/manner of articulation differences is apparent in the left superior temporal gyrus (STG) using electrocorticography (Mesgarani, Cheung, Johnson, & Chang, 2014), it is less clear that *dorsal* stream speech motor regions are involved in the perception of established, mature speech categories. Using a univariate fMRI approach, Pulvermüller et al. (2006) found differential activation in lip- and tongue-dedicated regions of the precentral gyrus in response to the syllables /p/ and /t/, which differ in place of articulation. These results were supported by findings from Correia, Jansma, and Bonte (2015), who used a multivoxel pattern analysis (MVPA) to identify brain regions that could classify neural responses to stimuli based on specific articulatory features independent of phonemic category (and subsequent acoustic variation). Activation patterns in pre-motor and motor areas (among other regions) could distinguish place and manner of articulation contrasts, even though the classifier was trained on responses to one phonological category (stop consonants) and tested on responses to another (fricatives). However, both Arsenault and Buchsbaum (2015) and Arsenault and Buchsbaum (2016) failed to find any dorsal stream regions that were sensitive to different articulatory dimensions of passively heard syllables in both univariate (attempting to replicate Pulvermüller et al.’s results) and multivariate analyses, calling into question the sensitivity of the dorsal stream to learned phonological contrasts.

As noted above, there is contradictory evidence that dorsal stream regions are sensitive to articulatory contrasts during speech perception. However, even in the studies that support the activation of the dorsal stream during speech processing, an alternative explanation exists: It is possible that what is being observed is IFG sensitivity to abstract sound categories at large, and not specifically the articulatory differences between sounds. That is, during speech perception, inferior frontal structures may not be coding articulatory differences between sounds (e.g., the difference in lip protrusion in the production of /i/ and /y/), but instead reflect the ultimate product of those gestures, which are two distinct phonetic categories. IFG sensitivity to speech categories has been established in a number of studies (Hutchison, Blumstein, Myers, 2008; Lee, Turkeltaub, Granger, & Raizada, 2012; Myers, 2007; Myers, Blumstein, Walsh, & Eliassen, 2009), but these studies do not disentangle the issue of articulatory codes versus abstract categories as the two are fundamentally confounded in established speech categories. In each of these previous studies, participants were presented with isolated speech sounds in their native language. These well-established perceptual categories may activate articulatory representations of the same sound by association rather than as a means to perceive the sound itself. That is, upon hearing “da,” one may automatically activate gestural codes for producing “da,” whether or not this articulatory information is necessary for retrieving the speech sound category. Further, as argued in the dual stream model, the dorsal stream may only be recruited during learning, when participants are learning the links between auditory and motor representations of sounds. Thus, to fully test the predictions of this model, it is necessary to present listeners with a situation in which a new speech category is acquired alongside an acoustically matched, inarticulable contrast in order to fully establish what dorsal stream activation may actually reflect.

Liebenthal, Binder, Spitzer, Possing, and Medler (2005) and Liebenthal et al. (2010) explored this idea by contrasting brain activation from familiar speech sounds (a continuum from /b/ to /d/) with that of an acoustically unfamiliar matched nonspeech continuum (created by spectrally inverting the first formant of the speech continuum). Although when participants had minimal exposure to the speech and nonspeech continua (Liebenthal et al., 2005), no dorsal stream differences emerged, after participants were trained on the same stimuli for four days, (Liebenthal et al., 2010), some dorsal stream recruitment was seen. That dorsal stream recruitment arises only during the learning process is consistent with the dual stream model, but paradoxically, dorsal stream engagement is not preferentially seen for speech (articulable) stimuli. Instead, several significant clusters in the left IFG, the bilateral postcentral gyrus, and the bilateral supplementary motor area display greater activation at posttest for a *nonspeech* continuum that could not be articulated by a human vocal tract. In a conjunction analysis, in which the neural changes as a result of training were compared across the speech and nonspeech continua, the left IFG was identified as sensitive specifically to learning in the nonspeech continuum. This provides further evidence that during learning, IFG activation more likely reflects the emergent category status of the nonspeech stimuli as listeners begin to form perceptual categories rather than a reflection of articulatory codes (Myers et al., 2009).

Desai, Liebenthal, Waldron, and Binder (2008), used a similar paradigm to Liebenthal et al. (2005), but critically substituted sine-wave speech versions of both the speech (/b/-/d/) and nonspeech continua (spectrally rotated /b/-/d/). Adopting the labels from this study, we will refer to them hereafter as “phonemic” and “nonphonemic,” respectively, to differentiate from the natural speech versions of these stimuli in previous studies. Sine-wave speech is created by taking the spectral center and power of each of the formants in a speech sound, and then creating a sine-wave tone at the same frequency and power (Remez, Rubin, Pisoni, & Carrell, 1981). The resulting sound contains approximately the same *distinctive* spectral information as the original speech sound but is not perceived as speech or speechlike until the listener is informed of the intended message, after which listeners often report being able to understand the message. These stimuli allowed the authors to control for familiarity and to manipulate the availability of articulatory information by revealing to participants midway through the experiment that one of the continua (the phonemic continuum) was made from familiar speech sounds. Dorsal stream recruitment was observed at posttest, where greater activation in the left IFG and the left precentral gyrus was seen for the phonemic continuum compared to the nonphonemic continuum. It seems possible that after being informed that the phonemic continuum was supposed to reflect two familiar speech sound categories, listeners began to recover some of the articulatory code used to create those sounds, and as a result the dorsal stream was recruited during learning. But again, this possibility does not exclude that neural activation reflects the emergence of two distinct categories, as perception of the phonemic continuum shifted from a continuous mode to a categorical mode during posttest.

### The Current Study

To investigate the role of the dorsal stream during the learning of new speech sounds, we attempted to equate the degree of proficiency with the two stimulus sets being learned, as well as to manipulate articulatory feasibility. While both variables have been manipulated independently in previous studies, to our knowledge no study has controlled for both of these variables simultaneously. We take a similar approach to Liebenthal et al. (2005, 2010) by introducing a set of articulable (nonnative speech) versus inarticulable (impoverished sine-wave speech) sounds. Like Desai et al. (2008), we used sounds that did not initially have established categories for our listeners, therefore requiring participants to learn both sets of sounds (unlike Liebenthal et al., 2005, 2010). However, in Desai et al., articulatory information is only indirectly implied in their phonemic continuum, as listeners must recover a phonological category from sine-wave speech. There is no guarantee that participants are activating articulatory representations of the source stimuli, and results could reflect listeners treating the sine-wave speech as inarticulable sounds but with a defined category structure. Therefore, we chose to use a non-native speech sound contrast to ensure that articulatory information is theoretically available, while the contrast is still as unfamiliar as the impoverished sine-wave speech contrast. Listeners are then trained to the same accuracy on both contrasts, after which two competing hypotheses can be investigated. First, following the logic of the dual stream model of speech perception, dorsal stream regions should only be recruited for auditory-motor integration during the learning of articulable sounds. Alternatively, dorsal stream regions (especially the IFG) may play a more general role in categorical decisions and will be equally recruited during the learning of both articulable and inarticulable sounds (so long as the two sounds are equally well learned).

The question at hand is especially suited to the use of MVPA (Haxby et al., 2001) because these techniques can provide a clue as to the nature of the information stored in different areas of the cortex. If the patterns of activation are distinctive enough to classify stimulus types, it implies that there is systematic neural representation for some property of that stimulus. Further, the differences in activation in response to a contrast like /b/ and /t/ may not be located in separate areas of the cortex, but may rather be better characterized by a distributed pattern of activation (including nonadjacent voxels), the kind of informational code that MVPA is sensitive to. By using signal decoding techniques, we ask not only whether articulatory versus auditory regions are recruited, but also whether the information content of those regions distinguishes between the two categories in each contrast.

We attempted to answer this question by using a multiday training study in which participants were trained to equal degrees of familiarity on an unfamiliar (but articulable) non-native phonetic contrast (the /i/ vs. /y/ contrast found in languages like French), as well as acoustically matched impoverished sine-wave analogues of the same contrast. After three days of training, participants then engaged in a passive fMRI task in which they heard tokens from the two continua they were trained on. A series of univariate and multivariate analyses were performed on the fMRI data to determine the neural architecture involved in the learning of new sounds, and how the articulatory feasibility of that sound impacts that architecture.

## MATERIALS AND METHOD

### Participants

Thirty-four undergraduate students were recruited from the University of Connecticut. All participants indicated that they were right-handed, monolingual native English speakers with normal hearing and no history of neurological or language disorders. Handedness and hearing abilities were self-reported by the participant, but not assessed in any other way. Eight participants were excluded (because of failure to complete all four sessions, *n* = 5, incomplete MRI data, *n* = 2, withdrawal, *n* = 1), leaving data from 26 participants (ages = 18–22 years, females = 16, males = 10) for analysis. Written informed consent was obtained from every participant in accordance with the guidelines of the University of Connecticut ethics committee. Participants were paid for their participation.

### Stimuli

#### Speech stimuli

The speech stimuli consisted of tokens taken from a seven-step vowel continuum from /i/ (front unrounded vowel, found in English in words like “leaf”) to /y/ (front rounded vowel found in languages such as French). This contrast was chosen because /y/ is not found in American English phonology and monolingual English speakers consistently struggle to discriminate these vowels without training (Levy & Strange, 2008; Darcy et al., 2012).

Natural speech tokens of /y/ and /i/ were produced by a male native speaker of Parisian French, and the /y/ token was then transformed into synthesized speech using the to KlattGrid function in Praat (Boersma & Weenink, 2020) to allow for precise control over acoustic properties. F1, F2, and F4 were fixed at the average values derived from the natural /y/ production, and F3 was systematically lowered in 7 equal Bark scale steps until the F3 value was equivalent to the naturally produced /i/ token’s F3 value, creating a continuum from /y/ to /i/. F5 was removed as it is uninformative to the contrast of interest. The synthesized /i/ and /y/ endpoints were submitted for pilot testing to five native speakers of French, who accurately identified the intended vowels and deemed them natural sounding. All tokens had a duration of 432 ms and were scaled to an intensity of 84 dB. Stimuli were delivered over headphones at a volume adjusted by the participant to a loud yet comfortable listening level.

#### Sine-wave stimuli

**Figure 1.**
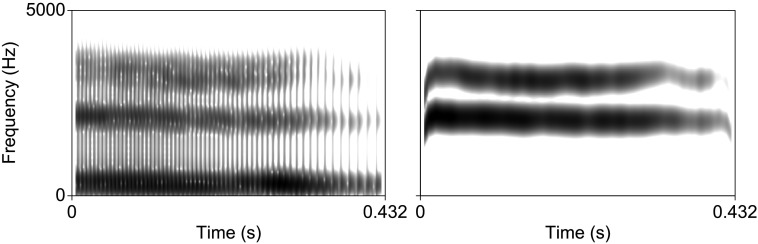
Spectrograms of the first step of the vowel (left) and sine-wave (right) continua.

Nonspeech acoustic analogues of the speech stimuli were created by transforming the vowel continuum described in the previous section into sine-wave speech. This was achieved using a Praat script created by Chris Darwin (available at http://www.lifesci.sussex.ac.uk/home/Chris_Darwin/Praatscripts/SWS). The resulting seven tokens were then stripped of the sine-wave equivalents of F1 and F4, leaving only the critical F2–F3 contrast in place to reduce the likelihood that they would be perceived as speech-like (see Figure 1). The resultant two-formant sine sweeps are thus acoustically matched to the diagnostic information in the vowel continuum (the frequency of F3), but critically during initial pilot testing (*N* = 11) they were never indicated to be perceived as speech (participants frequently described them as “chirps,” “bird sounds,” or “robot sounds”). The same pilot testing also revealed that a single session was sufficient for participants to reach near ceiling accuracy in categorizing sine-wave analogue tokens using the explicit perceptual fading training task described in the following sections (*M* = 97.7%, *SD* = 15%). All tokens had a duration of 432 ms and were scaled to an intensity of 80 dB.

### Experiment Schedule

**Figure 2.**
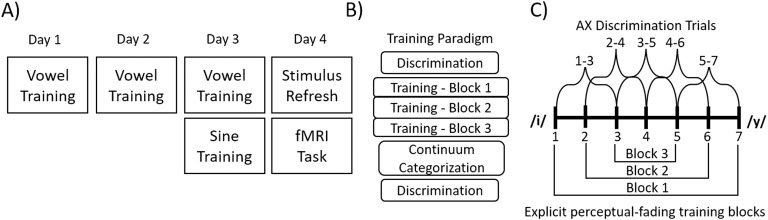
(A) Schedule of tasks completed over the course of the four days of training. (B) Schedule of the subtasks that comprised both the vowel and the sine-wave training sessions. (C) Overview of the stimulus pairs used in the discrimination (above the horizontal continuum line) and the explicit perceptual fading training (below the horizontal continuum line).

The study took place over four consecutive days (see Figure 2); on the first day, participants went through the informed consent process and completed the training paradigm for the vowel tokens. On the second day, participants returned and repeated training for the vowel tokens. On the third day, participants again repeated the vowel training followed by the sine-wave training paradigm. Finally, on the fourth day participants completed a short refresher on both stimulus sets, in which participants were refamiliarized with the correspondence between continuum sounds and the category they map on to, and then completed the fMRI task. The discrepancy in the number of training sessions between the vowel stimuli and the sine-wave stimuli was a result of earlier pilot testing, which indicated that it took participants one session to reach a threshold of at least 80% accuracy in the most difficult training block on the sine-wave stimuli, but three days to reach the same criteria for the vowel stimuli. The fMRI task was completed on a fourth day to allow for consolidation of the recently learned sine-wave stimuli, as sleep consolidation has been shown to impact non-native phonetic learning (Earle & Myers, 2015).

### Behavioral Training Regimen

The behavioral training paradigm consisted of three subtasks that were identical in structure across both Vowel and Sine training. OpenSesame 3.2.4 (Mathôt, Schreij, & Theeuwes, 2012) was used for stimulus presentation, and all responses were made via a Cedrus RB-840 response box. Participants wore Sony MDR-7506 headphones and were allowed to adjust volume to their most comfortable listening level. All response options were counterbalanced across participants.

#### Discrimination

At both the beginning and the end of the behavioral training paradigm, participants completed an identical AX discrimination task in which they were presented with a pair of tokens from the appropriate stimulus set that were either identical (e.g., step 1 vs. step 1) or two steps apart on the continuum (e.g., steps 1 vs. step 3; steps 4 vs. step 6) and asked to indicate as quickly and accurately as possible if the two sounds were the same or different. All pairs were presented both “forwards” (e.g., step 1 followed by step 3) or “backwards” (e.g., step 3 followed by step 1), with an intertrial interval of 500 ms. There were seven pair types where the two tokens presented were identical and ten pair types where the stimuli differed. Each pair was presented 11 times, for a total of 187 trials. Accuracy of response and reaction time were both recorded as outcomes of interest, and d′, an unbiased measure of sensitivity, was calculated from the participants’ responses. Trials in which participants failed to respond before the trial timed out (6,000 ms) were considered incorrect responses.

#### Perceptual fading training

Explicit training on the sound categories used a perceptual fading paradigm where participants were asked to map the two halves of the continuum to a different colored square. On each successive block, the acoustic difference between the two tokens that participants were asked to identify was reduced. To familiarize participants with the two categories they would be hearing, they were first shown a colored square and played 10 repetitions of the continuum endpoint token that would thereon be arbitrarily associated with that colored square (e.g., a red square would appear on the screen and the /i/ endpoint token would play).

In the first training block (labeled as “easy”), participants were asked to identify as quickly and accurately as possible which colored square corresponded to step 1 and step 7. On the second training block (labeled as “medium”), participants were asked to identify which colored square corresponded to continuum step 2 and continuum step 6. On the third training block (labeled as “hard”), participants were asked to identify which colored square corresponded to continuum step 3 and continuum step 5, the tokens that lay on opposing sides of the acoustic boundary. Notice that step 4, the midpoint of the continuum, was not presented during training.

Each block consisted of 60 trials (30 repetitions of each token), and participants were required to achieve an 80% accuracy threshold in order to ascend to the next block. If participants did not reach threshold by the end of the block, they would repeat that same block up to three times before being forced to move on to the subsequent block. Therefore, a given participant could experience between one and three repetitions of each block, depending on how quickly they advanced to more difficult blocks. Participants received explicit feedback during the task. Accuracy of response and reaction time were both recorded as outcomes of interest.

#### Continuum categorization

Following training, participants engaged in a two-alternative forced choice task in which they were asked to identify the colored square that corresponded to the sound being presented, with no feedback. There were 10 randomly presented repetitions of each token from the full seven-step continuum, for a total of 70 trials. The proportion of step 7 responses (which corresponded to the /y/_Vowel_ and /y/_Sine_ endpoints in the Vowel and Sine conditions respectively) per continuum step were then calculated. Trials in which participants failed to respond were omitted from analysis.

### Imaging Methods

#### fMRI design and procedure

Before entering the scanner, participants completed a brief task in which they were refamiliarized with the stimuli that would be presented during the fMRI task. Participants were first presented with a colored square while five repetitions of each of two tokens from that category were played. Following this, participants engaged in a two-alternative forced choice (2AFC) task in which they were asked to identify which colored square corresponded to the sound presented. Each of the four tokens were repeated 30 times, and this was repeated for both the Vowel tokens and the Sine tokens, leading to a total of 240 trials for the 2AFC task. Explicit feedback was provided during this task.

Participants were instructed to listen attentively and told that on some trials a stimulus would be presented more quietly than normal. When this happened, they should press any button on the MRI-safe response devices held in both hands. These catch stimuli consisted of a randomly chosen token from the eight Vowel and Sine tokens presented at 20 dB lower intensity. Participants completed a brief training during the structural scan acquisition to familiarize them with this task. Imaging data from catch trials were modeled in participant-level analyses but did not contribute to group-level analyses. Participants were on average quite accurate at detecting catch trials (*M* = 90%, *SD* = 30%). Stimuli were delivered by OpenSesame 3.2.4. The volume was set to a comfortable listening level as determined by each participant.

The fMRI experimental paradigm consisted of 10 runs, with run order randomized between participants. Trials within each run were presented in a fixed, pseudorandom order determined using the OptSeq2 tool (https://surfer.nmr.mgh.harvard.edu/optseq/). Steps 1, 3, 5, and 7 from both the Vowel and Sine continua were each repeated 15 times per run, along with 15 stimuli used for the catch task described in the previous paragraph, resulting in 150 presentations of each stimulus over the course of the experiment. An auditory stimulus appeared in all but 25 of the silent gaps during scanning, for an average intertrial interval of 2,390 ms. Each run consisted of 161 volumes.

#### Acquisition parameters

Anatomical and functional MRI data were collected with a 3-T Siemens Prisma scanner (Erlangen, Germany). High resolution three dimensional T1-weighted anatomical images were acquired using a multiecho magnetization prepared rapid gradient echo sequence (MPRAGE; repetition time [TR] = 2,400 ms, echo time = 2.22 ms, inversion time = 1,000 ms, 0.8 mm^3^ isotropic voxels, 320 × 320 matrix) and reconstructed into 208 slices. Functional echo planar images were acquired using an accelerated multiband sequence in ascending, interleaved order (52 slices, 2.5 mm thick, 2 mm^2^ axial in-plane resolution, 110 × 110 matrix, 220 mm^3^ field of view, flip angle = 62°) and followed a fast, sparse sampling design: Each functional volume was acquired with a 1,000 ms acquisition time, followed by 1,000 ms of silence during which auditory stimuli were presented (effective TR = 2,000 ms). Stimuli were always presented during the silent gap.

#### Univariate fMRI analyses

Data were analyzed using Analysis of Functional NeuroImages (AFNI; Cox, 1996). Preprocessing for the univariate analyses consisted of first transforming images from oblique to cardinal orientation, followed by motion correction using a six-parameter rigid body transform aligned with each participant’s anatomical data set, normalization to Talairach space (Talairach & Tournoux, 1988), and spatial smoothing with a 4 mm Gaussian kernel. Masks were created using each participant’s anatomical data to eliminate voxels located outside the brain. Individual masks were used to generate a group mask. Motion and signal fluctuation outliers were removed following standard procedures.

We generated time series vectors for each of the eight continua tokens (Vowel step 1, Vowel step 3, Vowel step 5, Vowel step 7, Sine step 1, Sine step 3, Sine step 5, Sine step 7) as well as the catch trials and any false alarm trials for each participant in each run. These vectors contained the onset time of each stimulus and were convolved with a stereotypic gamma hemodynamic function. The 10 condition vectors along with six additional nuisance movement parameters were submitted to a regression analysis. This analysis generated by-voxel fit coefficients for each condition for each participant.

For group level analysis, beta coefficients were submitted to a 2 × 3 analysis of variance (ANOVA) in AFNI (using 3dANOVA3). In the ANOVA, main effects of sound type (Vowel vs. Sine), continuum step (1 vs. 3 vs. 5 vs. 7), and their interaction were estimated.

#### Multivariate fMRI analyses

Data were preprocessed again using AFNI with a modified pipeline compared to the univariate analyses. Specifically, multivariate preprocessing consisted of first transforming images from oblique to cardinal orientation, followed by motion correction using a six-parameter rigid body transform aligned with each participant’s anatomical data set. No blurring or spatial normalization were performed until after the participant-level analysis to preserve fine-grained spatial information. The same 10 condition vectors from the univariate analyses along with six additional nuisance movement parameters were submitted to an individually modulated regression analysis (using the stim_times_IM flag in AFNI’s 3dDeconvolve), in which each trial for a condition is given its own amplitude estimation, yielding trial-wise beta coefficients (as opposed to run-wise beta coefficients, see Rissman, Gazzaley, & D’Esposito, 2004). The matrices from the output of the individually-modulated regression were then submitted to a least squares sum estimation using AFNI’s 3dLSS, in accordance with best practices for performing multivariate analyses on fast event-related fMRI designs (Mumford, Turner, Ashby, & Poldrack, 2012; Mumford, Davis, & Poldrack, 2014).

The beta coefficient output from 3dLSS was then used in an MVPA performed in MATLAB using the Decoding Toolbox (TDT; Hebart, Görgen, & Haynes, 2015). A linear support vector machine (SVM) classification was used with 3 voxel (6 mm) radius searchlight and a leave-one-run-out cross validation scheme, in which the SVM was trained on data from nine runs and tested on a tenth, held-out run. The regularization parameter for the SVM was set at C = 1. In a searchlight analysis, a roving sphere moves across the whole brain, and in each searchlight sphere an SVM was trained and tested (Kriegeskorte, Goebel, & Bandettini, 2006). The classification accuracy score was assigned to the central voxel of each searchlight. This yielded a participant-level classification accuracy map for each participant in their brain’s native space. The following classifications were performed: /i/ versus /y/ for vowel tokens (defined as step 1 and step 3 vs. step 5 and step 7, hereafter “/i/_Vowel_ vs. /y/_Vowel_”), and the analogous sine version (which we will hereafter refer to as “/i/_Sine_ vs. /y/_Sine_”).

In preparation for group-level analyses, participant-level classification maps were then normalized to Talairach space and blurred with a 4 mm Gaussian kernel. Following this, single-sample *t* tests against chance levels were performed for the /i/_Vowel_ vs. /y/_Vowel_ and /i/_Sine_ vs. /y/_Sine_ (using 3dttest++ in AFNI): *t* test output was masked by a group mask which only included voxels present in all 26 participants. In addition, a paired-samples *t* test was performed comparing the /i/_Vowel_ vs. /y/_Vowel_ and /i/_Sine_ vs. /y/_Sine_ decodings.

Cluster level correction for multiple comparisons was determined by running 10,000 iterations of Monte Carlo simulations on the group mask. Specifically, we used the -acf option in 3dFWHMx and 3dClustSim (AFNI) to estimate the spatial smoothness and generate voxel-wise and cluster-wise inferences. Data were corrected at a cluster level correction of *p* < 0.05 (voxel level threshold of *p* < 0.025, 149 contiguous voxels).

## RESULTS

### Behavioral Data Analysis and Results

#### Vowel training results

All the following behavioral analyses were conducted using the *R* packages afex and lme4 (Bates, Mächler, Bolker, & Walker, 2015; Singmann, Bolker, Westfall, Aust, & Ben-Shachar, 2020). Model selection was achieved by first creating and running all possible permutations of random effects structures, after which a backwards stepping selection procedure was applied to the models that converged (Matuschek, Kliegl, Vasishth, Baayen, & Bates, 2017). Output from the ANOVA table generated by the afex command “mixed” (run on the appropriate model selected from the earlier described procedure) is reported for all mixed-effects model analyses in this study. Residuals were inspected for each selected model with no deviations from normal encountered.

##### Perceptual fading training.

**Figure 3.**
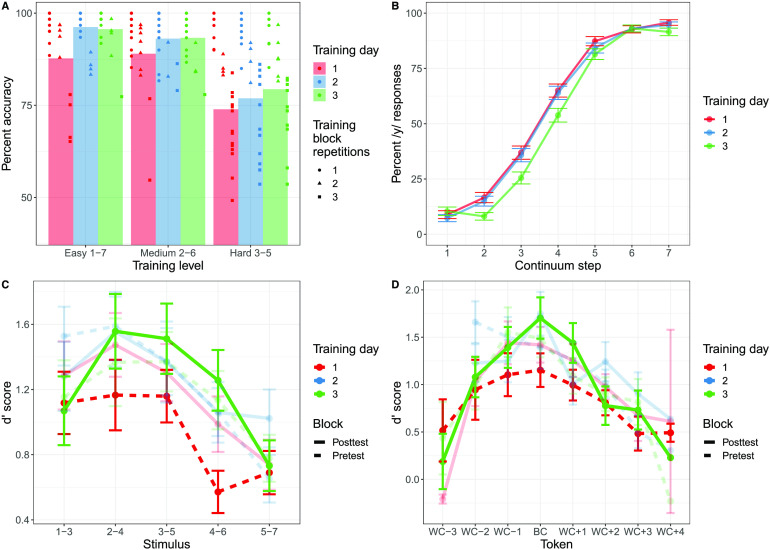
Behavioral data from the vowel training paradigm. (A) Accuracy on the explicit perceptual fading training task for the vowel stimuli as a function of training level difficulty. Each point indicates an individual participant’s performance, and the shape of the point indicates the number of times that participant repeated that block. (B) Responses to the continuum categorization task as a function of /i/_vowel_–/y/_vowel_ continuum step and training day. Error bars indicate standard error. (C) d′ scores from the AX discrimination task for the vowel stimuli across each stimulus as a function of pretest and posttest as well as training day. Error bars indicate standard error. (D) d′ scores from the AX discrimination task for vowel stimuli with respect to the participant-specific determination of the between-category (BC) and within-category (WC) tokens as a function of pretest and posttest as well as training day. Error bars indicate standard error.

On average, accuracy during training improved over the course of the three days (see Table 1, Figure 3A). Given the adaptive nature of the paradigm, participants completed varying repetitions of each level of difficulty (easy: *M* = 1.29 blocks, *SD* = 0.58; medium: *M* = 1.28 blocks, *SD* = 0.58; hard: *M* = 2.12 blocks, *SD* = 0.90), and as expected there was a strong negative correlation between the number of blocks completed and average accuracy on the explicit perceptual fading training (*r*(24) = −0.92, *p* < 0.0001), reflecting the fact that less proficient learners had to complete more blocks to progress through training.

**Table 1.** Accuracy during explicit perceptual fading training

Training	Easy (1–7)	Medium (2–6)	Hard (3–5)
Day 1: Vowel	*M* = 87.7% (*SD* = 33%)	*M* = 89% (*SD* = 31%)	*M* = 73.9% (*SD* = 43.9%)
Day 2: Vowel	*M* = 96.2% (*SD* = 19%)	*M* = 93.1% (*SD* = 25.3%)	*M* = 76.9% (*SD* = 42.2%)
Day 3: Vowel	*M* = 95.7% (*SD* = 20.3%)	*M* = 93.3% (*SD* = 25%)	*M =* 79.4% (*SD* = 40.5%)
Day 3: Sine	*M* = 93.7% (*SD* = 24.3%)	*M* = 94.9% (*SD* = 22.1%)	*M* = 83.9% (*SD* = 36.7%)

*Note*: Means (M) and standard deviations (*SD*) displayed.

Accuracy data during training was submitted to a generalized linear mixed-effects model using the *R* packages afex and lme4 (Bates, Mächler, Bolker, & Walker, 2015; Singmann, Bolker, Westfall, Aust, & Ben-Shachar, 2020). Model selection was achieved by first creating and running all possible permutations of random effects structures, after which a backwards stepping selection procedure was applied to the models that converged (Matuschek, Kliegl, Vasishth, Baayen, & Bates, 2017). Output from the ANOVA table generated by the afex command “mixed” (run on the appropriate model selected from the earlier described procedure) is reported for all mixed-effects model analyses in this study.

To examine the degree to which participants’ accuracy during vowel training increased over time, a generalized linear mixed-effects model was fit The selected model included fixed effects for training difficulty level, training day, and the interaction of training day and level, as well as by-subject random slopes and intercepts for the interaction between training difficulty level and training day. The model revealed a significant main effect of training difficulty (*χ*2 = 89.27, *p* < 0.0001) and a main effect of training day (*χ*2 = 7.45, *p* = 0.02), with higher accuracy on easier blocks and higher accuracy on successive days of training The interaction between training difficulty and training day did not reach significance (*p* = 0.62).

##### Vowel categorization posttest.

In general, participants were successful at identifying the endpoints of the continuum, with at least 90% accuracy on average across each of the three training days (see Figure 3B). Responses from the categorization posttest were transformed into a binary outcome variable and then submitted to a generalized linear mixed-effects model with a logit link function (also instantiated in the *R* package afex) to assess how categorization of the full continuum changed over the course of the three training days. *P* values were estimated using a likelihood-ratio test. The model selected included fixed effects for continuum step (centered) and training day, with by-subject random slopes and intercepts for continuum step, training day, and their interaction. Output from the model indicated a main effect of continuum step (*χ*2 = 44.15, *p* < 0.0001) and a main effect of training day (*χ*2 = 6.08, *p* = 0.05), indicating that participants’ /y/_Vowel_ responses increased as a result of the F3 manipulation across the continuum as expected, and that participants’ overall proportion of /y/_Vowel_ responses decreased with more experience with the continuum. The interaction was not significant (*p* = 0.82), indicating no significant change in the steepness of the categorization function over the three days of training.

##### AX discrimination task.

Discrimination data was first separated into pretest (the first discrimination task completed during that day’s training) and posttest (the final discrimination task following that day’s continuum categorization task), and “different” trials were collapsed such that discrimination token “step 1–step 3” was considered the same as “step 3–step 1.” Following this, d′ was calculated for each of the resulting five “different” tokens for each subject and training day. For the purposes of analysis, the token “3–5” was considered to be “between-category” (as step 4 of the continuum represents the most ambiguous token in terms of acoustics), and all other tokens were considered “within-category” (Figure 3C).

A linear mixed-effects model was then fit for participants’ d′ scores, with fixed effects of token type (between-category vs. within-category), training day, block (pretest vs. posttest) and their interactions, as well as by-subject random intercepts. A main effect of token type was found (*χ*2 = 12.30, *p* < 0.001), in that d′ scores were higher for the between-category token (step 3 – step 5) than the within-category tokens. In addition, a main effect of training day (*χ*2 = 3.09, *p* = 0.05) arose, indicating that d′ scores increased over the course of the experiment. All other main effects and interactions were nonsignificant.

Using the acoustic boundary to determine the between-category discrimination token ignores the fact that participants’ perceptual boundary did not necessarily correspond to the midpoint of the continuum. To remedy this concern, we estimated participant-relative perceptual boundaries and fit a linear mixed-effects model using the participant-relative determinations of the between-category and within-category tokens. The model selected contained fixed effects of token type (between-category vs. within-category), training day, block (pretest vs. posttest) and their interactions, as well as by-subject random intercepts. This model was then compared to the model previously fit using the acoustic boundary to determine whether a token was between-category or within-category. The model that used participant-relative boundaries had superior fit but yielded largely the same findings: a main effect of token type (*χ*2 = 38.76, *p* < 0.0001), a main effect of training day (*χ*2 = 5.19, *p* < 0.01), and a main effect of block (*χ*2 = 6.78, p < 0.01). All interactions were nonsignificant.

#### Sine training results

##### Explicit perceptual fading training.

Participants again completed varying repetitions of each level of difficulty (easy: *M* = 1.27 blocks, *SD* = 0.60; medium: *M* = 1.35 blocks, *SD* = 0.56; hard: *M* = 1.69 blocks, *SD* = 0.84), and there was a similarly strong negative correlation between the number of blocks completed and the average accuracy on the explicit perceptual fading training, *r*(7) = −0.86, *p* = 0.003.

To examine the efficacy of the single training session on identification of the two Sine tokens, a generalized linear mixed-effects model was performed. The model selected included fixed effects for training difficulty level and by-subject random intercepts (the maximal model for this analysis). The model revealed a significant main effect of training difficulty (*χ*2 = 102.98, *p* < 0.0001), indicating higher accuracy on easier blocks consisting of larger intervals on the continuum.

##### Sine AX discrimination task.

Discrimination data for the sine-wave stimuli was analyzed using the same procedure as the vowel stimuli. A linear mixed-effects model was then used for participants’ d′ scores, with fixed effects of token type (between-category vs. within-category), block (pretest vs. posttest), and their interaction, as well as by-subject random slopes and intercepts for all fixed effects and interactions.

**Figure 4.**
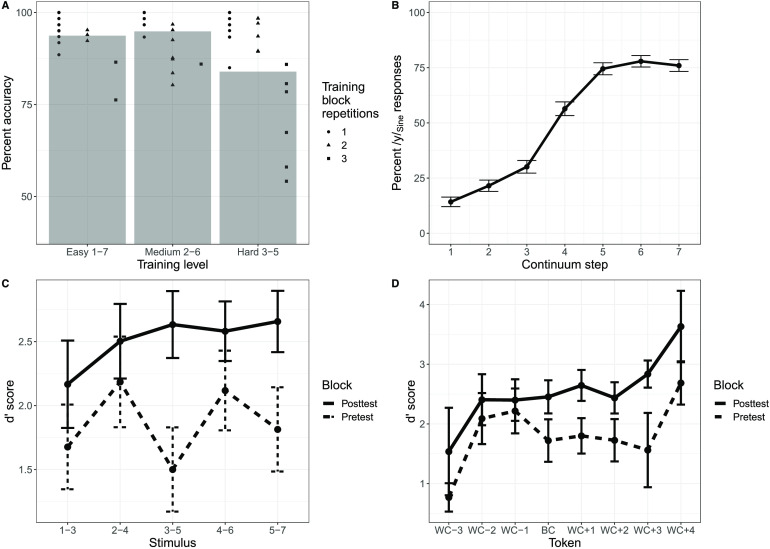
Behavioral data from the sine-wave training paradigm. (A) Accuracy on the explicit perceptual fading training task for the sine-wave stimuli as a function of training level difficulty. Each point indicates an individual participant’s performance, and the shape of the point indicates the number of times that participant repeated that block. (B) Categorization data as a function of the /i/_Sine_–/y/_Sine_ continuum step. Error bars indicate standard error. (C) d′ scores from the AX discrimination task for the sine-wave stimuli across each stimulus as a function of pretest and posttest. Error bars indicate standard error. (D) d′ scores from the AX discrimination task for sine-wave stimuli with respect to the participant-relative determination of the between-category (BC) and within-category (WC) tokens as a function of pretest and posttest. Error bars indicate standard error.

The model revealed that there was no significant difference in d′ between the between-category and within-category tokens (*p* = 0.26). However, there was a main effect of block, in which d′ scores overall increased from pretest to posttest (*F* = 9.43, *p* < 0.01). The interaction between token type and block was significant (*F* = 5.60, *p* < 0.05), indicating a larger increase in sensitivity at posttest for the between-category token than the within-category token. Recoding the data to use participant-relative determinations of the between-category token did not alter the results (Figure 4D).

#### Comparison of vowel and sine-wave behavioral performance

##### Explicit perceptual fading training.

Accuracy on the training task was comparable in sine-wave and vowel tokens, despite participants only having a single day of training (see Table 1, Figure 4A), which was expected from previous pilot testing. Data from the third day of training was analyzed with a generalized linear mixed-effects model with logit link function (the dependent variable, accuracy, was coded as binary value for each trial). Fixed effects of training difficulty level, stimulus type (Vowel vs. Sine), and their interaction, along with by-subject random slopes and intercepts for all fixed effects and their interactions were entered into the model. *P* values were estimated using a likelihood ratio test. The model output yielded a significant main effect of block (*χ*2 = 29.33, *p* < 0.0001), indicating that participants’ accuracy decreased as training difficulty increased, regardless of stimulus type, as well as a main effect of stimulus type (*χ*2 = 4.39, *p* = 0.04), in that accuracy was higher for the Sine stimuli compared to the Vowel stimuli. The interaction between block and stimulus type was nonsignificant (*p* = 0.07).

##### Categorization posttest.

Participants’ accuracy at identifying the endpoints of the Sine continuum was not as robust as with the Vowel continuum, but still reached at least 75% accuracy at each endpoint token (Figure 4B). Posttest categorization accuracy was analyzed using a generalized linear mixed-effects model where categorization of the Vowel (on the third training day) and Sine continua were compared. Fixed effects of continuum step, continuum type (Sine vs. Vowel), and their interactions, as well as random by-subject slopes and intercepts for all fixed effects and interactions were entered into the model. *P* values were estimated using a likelihood ratio test. There was a significant effect of continuum step (*χ*2 = 42.19, *p* < 0.0001), but the effect of continuum type was nonsignificant (*p* = 0.34). However, there was a significant continuum step by continuum type interaction (*χ*2 = 6.17, *p* = 0.01). Post hoc pairwise comparisons were performed using the *R* package lsmeans to investigate the source of the continuum step by continuum type interaction. There were significant differences (after Bonferroni correction) between the categorization of sine-wave and vowel stimuli at step 1 (*p* = 0.025), step 5 (*p* = 0.012), step 6 (*p* = 0.005), and step 7 (*p* = 0.004), which reflects the lesser degree of certainty at the endpoints for the sine-wave continuum.

Another metric of differences in the mode of categorization between the two stimulus sets (i.e., categorical perception vs. more continuous perception) is the slope of the categorization function. To this end, psychometric functions were fit to participants’ continuum categorization responses from the third day of training for both the sine-wave stimuli and the vowel stimuli using the package *quickpsy* (Linares & López-Moliner, 2016) in *R*. The slope of the psychometric function was used as a tool to quantify how strongly categorical an individual’s perception is, similar to the “categorical index” approach in Liebenthal et al. (2005, 2010), with the assumption that a steeper slope is among the behavioral hallmarks of more rigidly categorical perception. A linear mixed-effects model was then fit to the slopes of the psychometric functions, with a fixed effect of stimulus type (Vowel vs. Sine-wave) and random by-subject intercepts. There was no significant difference in slope values between stimulus sets (*F* = 0.55, *p* = 0.47).

**Figure 5.**
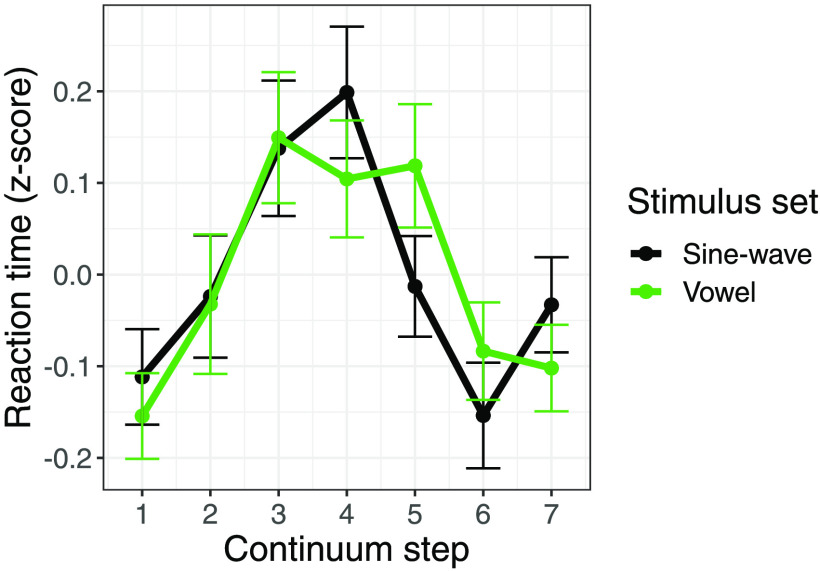
Reaction time (*z*-scored) in the continuum categorization task on the third day of training as a function of continuum step and stimulus set. Error bars indicate standard error.

Finally, to achieve converging evidence regarding the mode of categorization between the vowel and sine-wave stimuli, we also analyzed participants’ reaction times in the phonetic categorization task, as categorical perception is usually accompanied by slower responses for more ambiguous tokens in the center of a continuum than the clearer endpoint tokens (Pisoni & Tash, 1974). We again used responses only from the third day of training. To account for overall slower responses to the sine-wave stimuli compared to the vowel stimuli (sine-wave: *M* = 638.83 ms, *SD* = 577.35 ms, vowel: *M* = 473.64 ms, *SD* = 464.99 ms), we converted reaction times to *z*-scores. A linear mixed-effects model was fit to the *z*-scored reaction times, with main effects of stimulus type (Vowel vs. Sine-wave), continuum step (squared, to better reflect the quadratic shape of participants’ responses), and their interaction. The model selected included by-subject random slopes and intercepts for stimulus type and continuum step. There was no significant main effect of stimulus type (*p* = 0.93) or continuum step (*p* = 0.16), and critically no interaction between stimulus type and continuum step (*p* = 0.85), indicating that the pattern of responses for each step of the continuum, regardless of stimulus set, was not significantly different (see Figure 5).

##### AX discrimination task.

To evaluate the behavioral equivalence across stimulus sets, the AX discrimination data from the third day of training for the vowel stimuli was combined with the data from the sine-wave stimuli and analyzed using a linear mixed-effects model on the d′ scores. The model selected included main effects of token type (between-category vs. within-category), block (pretest vs. posttest), stimulus set (Vowel vs. Sine), and their interactions, as well as by-subject random slopes and intercepts for each main effect.

A main effect of token type was found (*F* = 4.53, *p* < 0.05), indicating higher overall sensitivity for between-category tokens than within-category tokens, as well as a main effect of block (*F* = 6.58, *p* < 0.05), indicating higher d′ scores at posttest than pretest, and a main effect of stimulus set (*F* = 21.68, *p* < 0.0001), with participants showing better overall sensitivity to sine-wave stimuli. In addition, a significant interaction of token type and stimulus set was found (*F* = 12.06, *p* < 0.001), as well as a significant interaction between block and stimulus set (*F* = 9.72, *p* < 0.01).

Post hoc pairwise comparisons on both interactions were again performed using the *R* package lsmeans with Bonferroni correction. The interaction between token type and stimulus set was driven by a significant difference between within-category and between-category tokens in the vowel data (*p* = 0.001), but not the sine-wave data (*p* = 0.34), while the interaction between block and stimulus set reflected an overall increase in d′ from pretest to posttest in the sine-wave condition (*p* = 0.0004) but no difference in the vowel condition (*p* = 0.51).

#### fMRI results

Imaging data from Sine and Vowel tokens were pooled in two analyses; a univariate approach to look for differences in activation magnitude, and a multivariate approach to investigate differences in activation patterns.

##### Univariate analysis.

**Figure 6.**
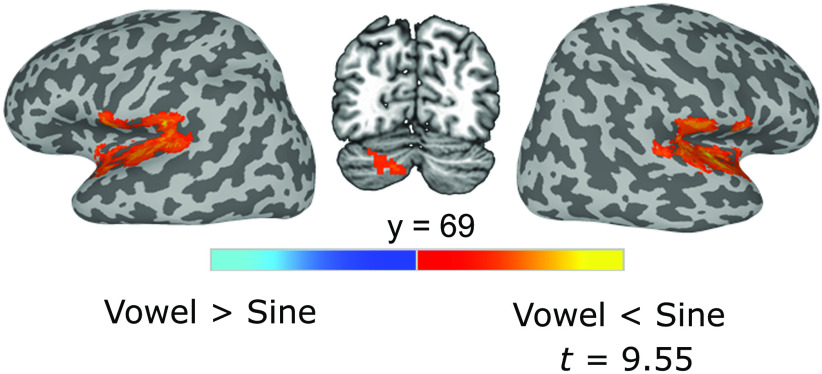
Regions that showed greater activation for sine-wave tokens than vowel tokens in the ANOVA. Clusters corrected at the voxel level of *p* < 0.025, with 149 contiguous voxels and a corrected threshold of *p* < 0.05.

Contrasting functional activation for vowel tokens compared to sine-wave tokens (Figure 6, Table 2) revealed three prominent clusters, all showing more activation for sine-wave tokens compared to vowel tokens. Significant activation differences were found in a large right hemisphere cluster in the STG extending into the Rolandic operculum, the insula, the temporal pole, and Heschl’s gyrus; an analogous left hemisphere cluster in the STG extending into Heschl’s gyrus, the Rolandic operculum, and the temporal pole and a smaller cluster in the left cerebellum.

**Table 2.** Results of univariate ANOVA comparing activation to Sine versus Vowel stimuli

Area	Cluster size (Voxels)	*x*	*y*	*z*	Maximum *t* value
*Sine > Vowel*					
L STG, L RO	1,807	−45	−11	6	7.85
R STG, R RO	1,777	47	−9	6	9.55
L Cere (Crus 2), L Cere (Crus 1)	150	−15	−69	−34	3.90

*Note*. L = left; R = right; STG = superior temporal gyrus; RO = Rolandic operculum; Cere = cerebellum. Clusters corrected at the voxel level of *p* < 0.025, with 149 contiguous voxels and a corrected threshold of *p* < 0.05.

To investigate how individual differences in proficiency with the two continua may have impacted neural results, a linear mixed-effects model (with fixed effects of stimulus type and continuum step, and by-subject random intercepts) was performed on the functional data using AFNI’s 3dLME. Accuracy on the hardest level of difficulty for the explicit perceptual fading training for both the vowel tokens and sine-wave tokens on the final day of training was selected as a covariate. After controlling for participants’ proficiency with the two continua, similar bilateral STG clusters emerged in which sine-wave tokens elicited greater activation than vowel tokens.

##### Multivariate analysis.

**Figure 7.**
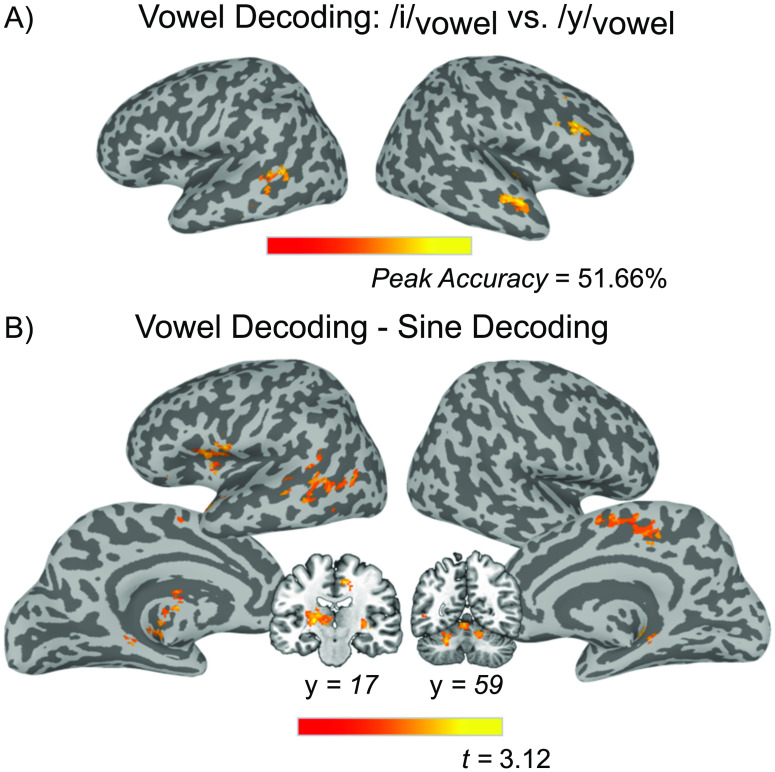
Results from the multivoxel pattern analyses. (A) Regions that showed significant classification of the two Vowel categories. Clusters corrected at the voxel level of *p* < 0.025, with 149 contiguous voxels and a corrected threshold of *p* < 0.05. (B) Output from the paired *t* test showing regions that classified Vowel categories significantly more accurately than Sine stimuli. The pop-out image displays cerebellar clusters and a thalamus cluster not rendered in surface image.

Participant-level classification accuracy maps from the searchlight MVPA analysis were submitted to single sample *t* tests against chance level (50%) to determine cortical regions that were able to discriminate category differences (e.g., all “A” tokens vs. all “B” tokens). For the decoding of the two vowel categories (/i/_Vowel_ vs. /y/_Vowel_), three significant clusters were found: (1) a cluster in the right middle temporal gyrus (MTG) extending into the right STG, (2) a cluster in the right IFG (specifically in the pars triangularis and the pars opercularis), and (3) a cluster in left MTG (see Table 3, Figure 7A). The same analysis for the sine-wave speech tokens, decoding /i/_Sine_ versus /y/_Sine_ did not yield any regions that met the threshold for significance, but voxel-level decoding results from the searchlight analysis contributed to the Vowel decoding–Sine decoding comparison below.

**Table 3.** Results of *t* tests performed on MVPA classification data

Area	Cluster size (voxels)	*x*	*y*	*z*	Peak accuracy
*/i/_vowel_ vs. /y/_vowel_ decoding*					
R MTG, R STG	200	47	−7	−12	51.64%
R IFG	181	49	19	30	51.66%
L MTG	159	−53	−43	2	51.47%
*/i/_Sine_ vs. /y/_Sine_ decoding*					
No significant clusters	–	–	–	–	–
Vowel decoding–Sine decoding					Maximum *t* value
L Thalamus	422	−21	−17	6	3.04
L Insula, L TP, L RO, L IFG	404	−33	−5	20	2.94
L MTG	288	−47	−59	6	2.87
R SMA, R MCC, L SMA	227	9	−17	44	2.93
R Cere (VI), R Cere (IV-V)	191	15	−53	−22	2.97
L Cere (IV-V), Cere Verm (4/5)	186	−3	−57	−8	3.18
R HC, R Put	171	27	−29	0	3.06
L Cere (VI), L Cere (Crus 1)	162	−23	−55	−26	2.93

*Note*. L = left; R = right; MTG = middle temporal gyrus; STG = superior temporal gyrus; IFG = inferior frontal gyrus; TP = temporal pole; RO = Rolandic operculum; SMA = supplementary motor area; MCC = middle cingulate cortex; Cere = cerebellum; HC = hippocampus; Put = putamen; Cere Verm = cerebral vermis. Clusters corrected at the voxel level of *p* < 0.025, with 149 contiguous voxels yielding a corrected threshold of *p* < 0.05.

Next, we contrasted the sine-wave and vowel decoding maps to identify regions that differed significantly in decoding performance for the two stimulus sets. Participant-level classification accuracy maps from the sine-wave and vowel decoding analyses were submitted to a paired-samples *t* test to identify regions that showed significantly different decoding performance for Vowel category differences than for Sine category differences. All such clusters showed superior decoding of Vowel contrasts and included a cluster that extended from the left insula into the left temporal pole and the left IFG, as well as another cluster in the left MTG (Table 3, Figure 7B).

## DISCUSSION

### Behavioral Results

The intent in this study was to train participants to equal proficiency on two novel contrasts, a non-native vowel contrast and an acoustically matched sine-wave contrast. There are many metrics that can be considered when evaluating the degree to which we were successful in doing so; overall accuracy during training reflects the degree to which contrasts were learnable in the moment, the slope of the categorization curve reflects the precision with which participants separated the categories, and differences in discrimination performance (in particular spikes in discriminability of tokens that cross the category boundary) reflect the emerging “categorical” status of the sound category. It is notable that these metrics do not necessarily pattern together, even in well-developed native language categories. For instance, several studies have found no relationship between the categorization function and discrimination performance (Gerrits & Schouten, 2004; Hary & Massaro, 1982; van Hessen & Schouten, 1999; Schouten, Gerrits, & van Hessen, 2003).

On balance, participants showed successful learning of both contrasts: Participants’ ability to both identify and discriminate between the two categories in each continuum increased with training, and ultimately, performance on the training task, the slope of the category boundary, and the shape of the reaction time function were not significantly different between the two continua. The continua differed, however, in the degree of “acquired distinctiveness” in the discrimination data, with the vowel stimuli showing marked increases in sensitivity to between-category tokens, while sine-wave stimuli showed much higher overall discriminability, but no obvious peak for between-category tokens. This pattern may reflect qualitative differences in the way that speech versus nonspeech categories emerge, with speech sound categories more likely to have perceptual warping within the category, a classic hallmark of native language speech categories. Below we consider our results in the context of other literature on speech and nonspeech category learning.

The vowel contrast in particular showed a behavioral pattern that qualitatively resembles nativelike perception (i.e., an increased sensitivity across category boundaries, a decreased sensitivity within category boundaries, a sharp categorization curve, and an increased reaction time to near-boundary decisions (Liberman, Harris, Hoffman, & Griffith, 1957; Pisoni & Tash 1974). On the last day of training, d′ values from the AX discrimination task for within-category tokens, (e.g., 1–3 or 5–7) were much lower than on the first day of training, whereas d′ scores increased for the tokens most often containing participants’ perceptual boundary (2–4 and 3–5). These results are congruent with previous studies of native English speakers’ perception of the front-rounded vowel /y/, which shows that listeners will initially assimilate /y/ to the American English vowel /i/. Nonetheless, this contrast is ultimately learnable to a high level of proficiency (Levy & Strange, 2008; Levy, 2009).

Sine-wave versions of speech stimuli are not automatically mapped to speech analogues. In our study, we removed F1 and F4 to prevent listeners from reconstructing the formants for the /i/ and /y/ vowels and thus retrieving those speech categories. Studies of the perception of sine-wave speech continua have found that participants’ responses in identification tasks were either random or largely continuous (Grunke & Pisoni, 1982; Johnson & Ralston, 1994, exp. 1; Desai et al., 2008), though a sine-wave speech version of a vowel continuum was perceived categorically regardless of whether the listeners interpreted them as speech or nonspeech (Johnson & Ralston, 1994, exp. 2). As discussed above, the sine-wave behavior is betrayed by one finding that suggests that the sine-wave category was more poorly learned (or less fully elaborated) than the vowel stimuli, namely a lack of an obvious peak in the discrimination function around the category boundary. However, the significant interaction present in the sine-wave AX discrimination analysis, wherein there was a greater increase in sensitivity for the between-category token than the within-category tokens at posttest, does point to an emerging pattern for acquired distinctiveness. Without additional exposure, this point will remain speculative as there is a lack of converging research on discrimination performance for sine-wave speech after multiple days of training.

### fMRI Results

In general, greater activation for the sine-wave tokens in the univariate analysis points to the possibility that they were being processed less efficiently by a general auditory, and not a speech-specific, mechanism. A broad scale contrast of areas where sine-wave tokens elicited greater activation than vowel tokens revealed bilateral clusters in STG, similar to the findings in Liebenthal et al. (2010), where nonspeech stimuli showed greater activation than speech stimuli at posttest in STG. This could be due to the fact that the sine-wave tokens were still more novel and participants had less of an opportunity to habituate to them. However, our results replicate Liebenthal et al. (2010), where participants were trained over the course of four sessions on their nonspeech stimuli, suggesting that mere lack of exposure is not the only driving factor behind the sine-wave vs. vowel activation differences. Given the broad scale of dimensions that vary between the two stimulus sets (e.g., articulatory information or acoustic complexity), it is difficult to draw conclusions about the driving force of these neural differences using only univariate methods.

#### Regions identified in vowel decoding analysis are outside of dorsal stream

As discussed earlier, the dual stream model posits that the dorsal stream is recruited during the learning of speech sounds, where it serves as an auditory-motor integration mechanism. Evidence for this role has been difficult to ascertain given that many previous studies have used stimuli where only the inarticulable stimuli were unfamiliar (e.g., Liebenthal et al. 2005, 2010). To more conclusively answer this question, we trained participants on an unfamiliar vowel contrast, and used multivariate decoding analyses to investigate which brain regions could represent the differences between these sounds. Any region that can decode these newly learned vowel categories could do so by virtue of access to (1) articulatory differences (lip rounding differences that listeners infer or extract from the auditory input), (2) abstract phonological categories (/i/ vs. /y/), or (3) the acoustic differences between the two sounds (F3 is higher in /y/).

We found that the right MTG and the right STG were sensitive to vowel category information. Crucially these areas are not included in the dorsal stream or implicated in speech-motor processing but *have* been identified in previous imaging studies of non-native phonetic learning (Callan et al., 2003; Wong, Perrachione, & Parrish, 2007). Additionally, another significant cluster was found in the right IFG, which along with the right MTG, has been implicated as playing a role in perceptual learning for speech, as Myers and Mesite (2014) found it to be sensitive to changes in representation of an /s/-/∫/ contrast in response to a talker-specific artificial accent. The right IFG has also been shown to be sensitive to nonlinguistic acoustic processing, like pitch (Wang, Sereno, Jongman, & Hirsch, 2003), as well as to play a role in non-native phonetic learning (Myers & Swan, 2012). Thus, we take it as more likely that decoding sensitivity in these right hemisphere regions is based upon differential representations of the abstract categories of /i/_Vowel_ and /y/_Vowel_ and not the differences in articulation between them (or simply the acoustic differences). The only left hemisphere region that successfully decoded the two vowel categories was the left MTG, and the cluster overlaps strongly with the phonological network described as part of the ventral stream (Hickok & Poeppel, 2007). As such, our preferred interpretation of this pattern is that the left MTG likely represents phonological category differences between /i/ and /y/. The failure to find evidence for dorsal stream representation of different speech sounds during phonetic learning, when it is predicted by the dual stream model, suggests that articulatory information is either coded in areas not involved in speech-motor gestures (e.g., the right MTG and the STG) along with phonological representations, or represented at a level that our multivariate analysis is not sensitive to, or else simply not recruited during this task.

#### Dorsal stream identified only when vowel decoding is contrasted against sine-wave decoding

It is possible the sensitivity to newly learned vowel categories reflects acquisition of any new auditory categories, and is not speech-specific. In order to differentiate these possibilities, we compared classification results from the /*i*/_*Vowel*_ vs. /*y*/_*Vowel*_ and the /*i*/_*Sine*_ vs. /*y*/_*Sine*_ decoding maps. The resulting statistical map indicates regions that better decoded the two different vowel categories than the two sine-wave categories, or put differently, regions that are sensitive specifically to auditory contrasts that differ in articulation. The critical comparison of interest is whether dorsal stream regions show discriminable patterns of activation *only* during the learning of articulable sounds (Vowel) but not nonarticulable sounds (Sine), as would be predicted by the conception of the dorsal stream as an auditory-motor integration mechanism. Several dorsal stream regions emerged from this analysis; the left IFG and the left insula, the bilateral supplementary motor areas, and a left MTG extending into posterior STG (see Figure 7B) all showed superior decoding of vowel tokens compared to the sine-wave analogues.

These results are consistent with several transcranial magnetic stimulation (TMS) studies of the role of motor regions in speech perception, in which “artificial lesions” of speech motor regions (i.e., primary and pre-motor cortices) lead to changes in discriminating or identifying *native language* speech sounds, indicating a causal role of the articulatory regions during speech perception (Rogers, Möttönen, Boyles, & Watkins, 2014; Möttönen & Watkins, 2009; Krieger-Redwood, Gaskell, Lindsay, & Jefferies, 2013; Sato, Tremblay, & Gracco, 2009; Meister et al. 2007; Smalle, Rogers, & Möttönen, 2015). However, these studies have not necessarily addressed the dorsal stream as circuit but have instead focused on its constituent regions one at a time. For instance, Krieger-Redwood et al. (2013) showed that stimulation of dorsal stream regions affected phonetic decisions but not performance, on a more naturalistic task where listeners had to match a word to a sample picture. Murakami, Kell, Restle, Ugawa, and Ziemann (2015) applied TMS simultaneously to the left pre-motor cortex and the left IFG (two of the three regions of the dorsal stream) and found decrements in phonological processing only in noisy conditions, which suggests that the dorsal stream may only be brought online during perception when stimulus identity is unclear. In our study, it is possible that the dorsal stream is recruited when the newly learned vowel /y/ must be distinguished from similar English vowels already in the repertoire. By contrast, the sine-wave stimuli most likely do not assimilate to any existing auditory categories, and thus their identity is more easily resolved.

The results from the Vowel decoding–Sine-wave decoding analysis do provide support for the dual stream model’s conception of the dorsal stream as an auditory-motor integration mechanism, but we hesitate to conclusively accept this interpretation. As we will discuss in the next section, no brain regions significantly decoded sine-wave stimuli, which means that the Vowel decoding–Sine-wave decoding analysis is displaying areas that have higher accuracy in the vowel decoding, but nevertheless do not reach significance in the vowel decoding alone (there was essentially no overlap between the map generated by the Vowel decoding–Sine-wave decoding analysis and the map generated by the vowel decoding alone beyond the left MTG). To that end, we believe the results from the vowel decoding, which do not involve any dorsal stream regions, sufficiently address the original motivation of this study. Based upon these results, future research should step back and more thoroughly examine if the dorsal stream is truly involved in phonetic learning at all.

Another potential issue with the Vowel decoding–Sine-wave decoding lies in the present study’s ability to completely control the equivalence of category structure across the vowel and sine-wave stimuli. As our motivating goal was to investigate whether dorsal stream recruitment in previous studies reflected auditory-motor integration or simply the formation of new auditory categories, it was necessary to have two sets of stimuli that differed only on the dimension of articulatory feasibility. As discussed above, although our stimuli were equated in terms of participants’ success on the training task, that equivalent accuracy may have led to qualitative differences in the shape of the emerging categories. Specifically, by one metric (discrimination) our sine-wave stimuli differed from our vowel stimuli in their degree of categoricity. Therefore, it is possible that the regions found in the Vowel decoding–Sine-wave decoding could reflect differences in the emergence of categorical perception and not just articulatory information. Future research will need to more strongly control for category structure to more precisely equivalate between speech and nonspeech sounds.

#### Gradient neural representations of sine-wave stimuli may have impeded decoding

The sine-wave decoding analysis did not uncover any brain regions that reliably represented the two sine-wave categories. This is a bit of a puzzle given strong behavioral evidence that participants were able to distinguish the categories. One explanation for the failure to find neural sensitivity to sine-wave tokens appeals to the degree to which these stimuli showed “acquired equivalence” within the sound category. In speech processing, acoustic cues to speech sounds are initially encoded continuously at the neural level (Blumstein, Myers, & Rissman, 2005; Frye et al., 2007; Toscano, McMurray, Dennhardt, & Luck, 2010), then categorical structure is imposed on them quickly and neural responses begin to reflect phonological category membership early in processing (Toscano, Anderson, Fabiani, Gratton, & Garnsey, 2018). We speculate that by contrast, nonspeech categories such as the sine-wave speech tokens used in the current study may continue to be represented in a graded fashion at the neural level. Because our analysis favored grouping of two more perceptually distinct sine-wave stimuli to reflect a single category (e.g., steps 1 and 3 for /i/_Sine_), this could have created a more difficult classification problem for sine-wave tokens. Submitting just the endpoints of the sine-wave continuum (step 1 and step 7) to the same decoding analysis yielded a significant cluster in the right MFG. Notably, this analysis suffers from less power than that reported in the main body of the text, using only half of the tokens in the decoding analysis. This finding contrasts with a previous multivariate fMRI study of trained nonspeech novel auditory categories that found that bilateral primary auditory cortex activation could successfully classify the two categories (Ley et al., 2012).

On the topic of classifier accuracy, observed accuracy at the group levels was numerically low, but statistically reliable. For the goals of this experiment, maximizing accuracy of the classifier is not of primary importance; significantly above-chance findings indicate that there are structured neural patterns in response to different stimuli, and using accuracy levels as a measure of the size of this effect is a faulty assumption. For a detailed explanation of the differences between MVPA for interpretation versus prediction, see Hebart and Baker (2018).

#### Subcortical regions implicated only in learning of speech sounds

Speech sound processing, especially during learning and adapting to speech sound variants, may not be limited to cortical regions. In the present data, better decoding for the vowel contrast compared to the sine-wave contrast was found in the left thalamus. Previous work has observed that the thalamus is sensitive to human speech sounds: Dehaene-Lambertz et al. (2005) found that the thalamus was generally more active for speech rather than sine-wave speech analogues in humans, while Kraus et al. (1994) found that the guinea pig thalamus is sensitive to complex spectral differences between human speech sounds even when animals were not exposed to any kind of training on these sounds. However, more relevant to this goal of this study is the relationship between the thalamus and articulatory information; neuropsychological investigations have found that damage to the thalamus often yields difficulties with the articulation of speech sounds (Jonas, 1982; Wallesch et al., 1983), and our results suggest that during the learning of new speech sounds, the thalamus may be representing the articulatory codes that will later be used for production, similar to the role of the dorsal stream in the dual stream model.

More broadly, there has been increasing interest in the contribution of subcortical brain structures in experience-dependent plasticity in the auditory system (Chandrasekaran, Skoe, & Kraus, 2014), and it is suggested that the cerebellum is involved in adaptation to alterations in speech through its functional connectivity to cortical language areas (Guediche, Holt, Laurent, Lim, & Fiez, 2015). In the current study, the significant right cerebellar clusters may reflect the engagement of this language plasticity network (similar to Guediche et al., 2015) as a result of the nascent formation of a speech sound category. Language related cerebellar function is heavily right lateralized, while the left cerebellum, especially lobule VI and Crus 1, are implicated as having a role in executive functioning. Left cerebellar engagement in this study could reflect a decision-making process about category membership for the vowel contrast (for a review, see Stoodley & Schmahmann, 2009).

### Conclusion

In sum, the MVPA results from the present experiment provide support for the interpretation that dorsal stream regions are recruited during the learning of articulable sounds only, a question that has been obscured in previous studies due to stimuli not being equated on proficiency or articulatory feasibility. We did find evidence for dorsal stream recruitment when looking for regions that could preferentially discriminate category membership for articulable contrasts (our vowel contrast) rather than inarticulable contrasts (our sine-wave contrast); however, differences in category structure between the articulable and inarticulable contrasts (in that the former was perceived more categorically by some metrics than the latter) also allow for the possibility that this recruitment reflects sensitivity to category structure and not articulatory feasibility. However, these regions did not appear when examining decoding of the two articulable vowel sounds alone. Instead, a series of regions outside of the dorsal stream previously implicated in non-native phonetic learning could successfully classify the two vowel categories. Future work will need to address the interplay between recruitment of the dorsal stream in the initial stages of phonetic learning and the contribution of right hemisphere cortical regions.

## FUNDING INFORMATION

Emily B. Myers, National Institute on Deafness and Other Communication Disorders (http://dx.doi.org/10.13039/100000055), Award ID: R01DC013064. James Magnuson, National Science Foundation (http://dx.doi.org/10.13039/100000001), Award ID: 1735225.

## AUTHOR CONTRIBUTIONS

Emily Myers proposed the original motivation and design of the experiment. Emily Myers and David Saltzman created the materials and final design. David Saltzman collected the data and performed all analyses. David Saltzman wrote the initial draft of the manuscript, with additions and revisions by Emily Myers. David Saltzman responded to reviewers’ comments with additions from Emily Myers.
